# Matrix stiffness affects tumor-associated macrophage functional polarization and its potential in tumor therapy

**DOI:** 10.1186/s12967-023-04810-3

**Published:** 2024-01-21

**Authors:** Jiaqiang Xiong, Rourou Xiao, Jiahui Zhao, Qiuyan Zhao, Manwen Luo, Feng Li, Wei Zhang, Meng Wu

**Affiliations:** 1 Department of Obstetrics and Gynecology, Zhongnan Hospital of Wuhan University, Wuhan, 430071 China; 2 Department of Medical Genetics, School of Basic Medical Sciences, Wuhan University, Wuhan, 430071 China; 3 Hubei Provincial Key Laboratory of Allergy and Immunology, Wuhan, 430071 China; 4 Department of Obstetrics and Gynecology, Tongji Hospital, Tongji Medical College, Huazhong University of Science and Technology, Wuhan, 430032 China

**Keywords:** Matrix stiffness, Macrophage polarization, Mechanoreceptors, Mechanotransducers, Cancer therapy

## Abstract

The extracellular matrix (ECM) plays critical roles in cytoskeletal support, biomechanical transduction and biochemical signal transformation. Tumor-associated macrophage (TAM) function is regulated by matrix stiffness in solid tumors and is often associated with poor prognosis. ECM stiffness-induced mechanical cues can activate cell membrane mechanoreceptors and corresponding mechanotransducers in the cytoplasm, modulating the phenotype of TAMs. Currently, tuning TAM polarization through matrix stiffness-induced mechanical stimulation has received increasing attention, whereas its effect on TAM fate has rarely been summarized. A better understanding of the relationship between matrix stiffness and macrophage function will contribute to the development of new strategies for cancer therapy. In this review, we first introduced the overall relationship between macrophage polarization and matrix stiffness, analyzed the changes in mechanoreceptors and mechanotransducers mediated by matrix stiffness on macrophage function and tumor progression, and finally summarized the effects of targeting ECM stiffness on tumor prognosis to provide insight into this new field.

## Introduction

The extracellular matrix (ECM) is the basis of maintaining tissue structure and organ homeostasis [1]. It is also a vital component of the tumor microenvironment (TME), supporting the initiation, progression, and invasion of tumors [2]. ECM is mainly composed of collagen, fibrin, elastin, fibronectin, glass adhesin, glycoproteins and other matrix proteins, which determine the stiffness and elasticity of tumor tissue [3]. Tissue stiffness is dynamically regulated and remodeled by the synthesis and degradation of ECM proteins. Degradation of peripheral ECM components by matrix metalloproteinases (MMPs), cathepsin, and hyaluronidase is an important pathogenic mechanism of the dynamic regulation of ECM structure [4]. During tumor progression, ECM degradation releases a suite of growth factors and cytokines that induce tumor cell growth, angiogenesis and inflammation [5]. Moreover, ECM degradation is accompanied by the deposition of different tumor-specific ECMs, finally contributing to an increase in tissue density and rigidity[6]. Generally, the matrix stiffness of soft tissues, such as the brain, liver, colon and fat tissue, is typically less than 10 kPa; however, in diseased conditions, such as fibrosis and solid tumors, the mean stiffness value can exceed 20 kPa and 50 kPa, respectively [7]. Recently, accumulating evidences demonstrate that increased matrix stiffness is associated with poor clinical outcomes [8, 9], and ECM stiffness is widely recognized as a new hallmark in solid tumors [10, 11]. Therefore, targeting ECM stiffness is emerging as a potential option for cancer therapy.

Tumor-associated macrophages (TAMs) are the most prominent innate immune cells in the TME, accounting for approximately 50% of CD45^+^ cells in the tumor tissue, with heterogeneity and plasticity ranging from antitumor to protumor [12]. There are two main sources of TAMs: (I) monocytes produced by myeloid progenitors of bone marrow, including classical Ly6C^+^ monocytes and nonclassical Ly6C^−^ monocytes. These monocytes leave the blood circulation and enter tumor tissue to differentiatiate into macrophages [13]. (II) Early embryonic source: yolk sac or fetal liver. Macrophages become tissue-resident macrophages, which are present in various healthy tissues and participate in cancer growth and metastasis [14]. After monocytes dissociate from the bone marrow, monocytes are recruited to the TME by chemokines such as CCL2, CCL5 and CXCL12, which are produced by cancer cells in the early stages of tumorigenesis [15]. At present, the most widely used traditional classification of TAMs is the dual classification, that is, the antitumor concomitant pro-inflammatory M1 phenotype (classical activation) and the protumor concomitant anti-inflammatory M2 phenotype (alternative activation) [16–18]. For instance, bacterial products such as lipopolysaccharides and pro-inflammatory factors such as IFN-γ can induce M1 macrophages to produce inflammatory factors and chemokines (including IL-1, IL-6, IL-12, TNF-α, CCL3, CCL5, CXCL8, CXCL9, and CXCL10), with the upregulation of iNOS as well as CD80, CD86, and MHCII [19]. While certain signaling pathways, such as STAT6 and PPARγ, along with parasitic infections, have the capacity to induce M2 macrophages to produce anti-inflammatory cytokines (including IL-4, IL-10, and IL-13), as well as express markers such as Arg1, TGF-β, CD163, CD206, VEGF, and MMPs, these signaling molecules accelerate TME remodeling, angiogenesis and tumor growth [20]. In addition, some researchers have added an additional subcategory to M2 TAMs, such as M2a, M2b, M2c and M2d, due to the complexity of TAMs [21]. Furthermore, single-cell sequencing analysis revealed that TAM subsets can express both M1- and M2-related genes with complicated phenotypes, which indicates a state of phenotypic transition between M1 and M2 TAMs [22]. In fact, M1 and M2 represent only the extremes of the more complex and continuum spectrum of macrophages activation states, and macrophages are an extremely plastic cellular population depending on tumor context and stage of disease [23].

In the early stage of tumorigenesis, TAMs are mainly the M1 phenotype, and with the development of the tumor, TAMs are mainly the M2 phenotype with a significant immunosuppressive function in the TME [24]. Besides the hypoxic microenvironment, tumor cells, and fibroblasts, matrix stiffness represents a significant factor driving macrophage polarization toward the M2 phenotype. Furthermore, accumulating evidence demonstrates that the ECM is not only the core component of tumor tissue but also has extensive and complex biochemical and biomechanical contact with TAMs in the cancer microenvironment, supporting the proliferation, progression, and invasion of cancer cells [25]. Given that matrix stiffness affects the phenotype and function of TAMs and is often associated with a poor clinical prognosis, a better understanding of the mechanisms underlying the regulatory network between matrix stiffness and macrophages is critical for targeted therapy [26].

Matrix stiffness or mechanical stimulation first senses mechanical changes through the mechanoreceptors on the plasma membrane and then transmits mechanical signals through mechanically related molecules in the cytoplasm, ultimately modulating nuclear transcription or the function of macrophages. Therefore, in this review, we discuss the mechanoreceptors and the corresponding key mechanical molecules of macrophages regulated by matrix stiffness or mechanical stimulation and summarize ECM stiffness-based targeted cancer therapy as well as the shortcomings and challenges in this field.

## Matrix stiffness and macrophage polarization

**Fig. 1:**
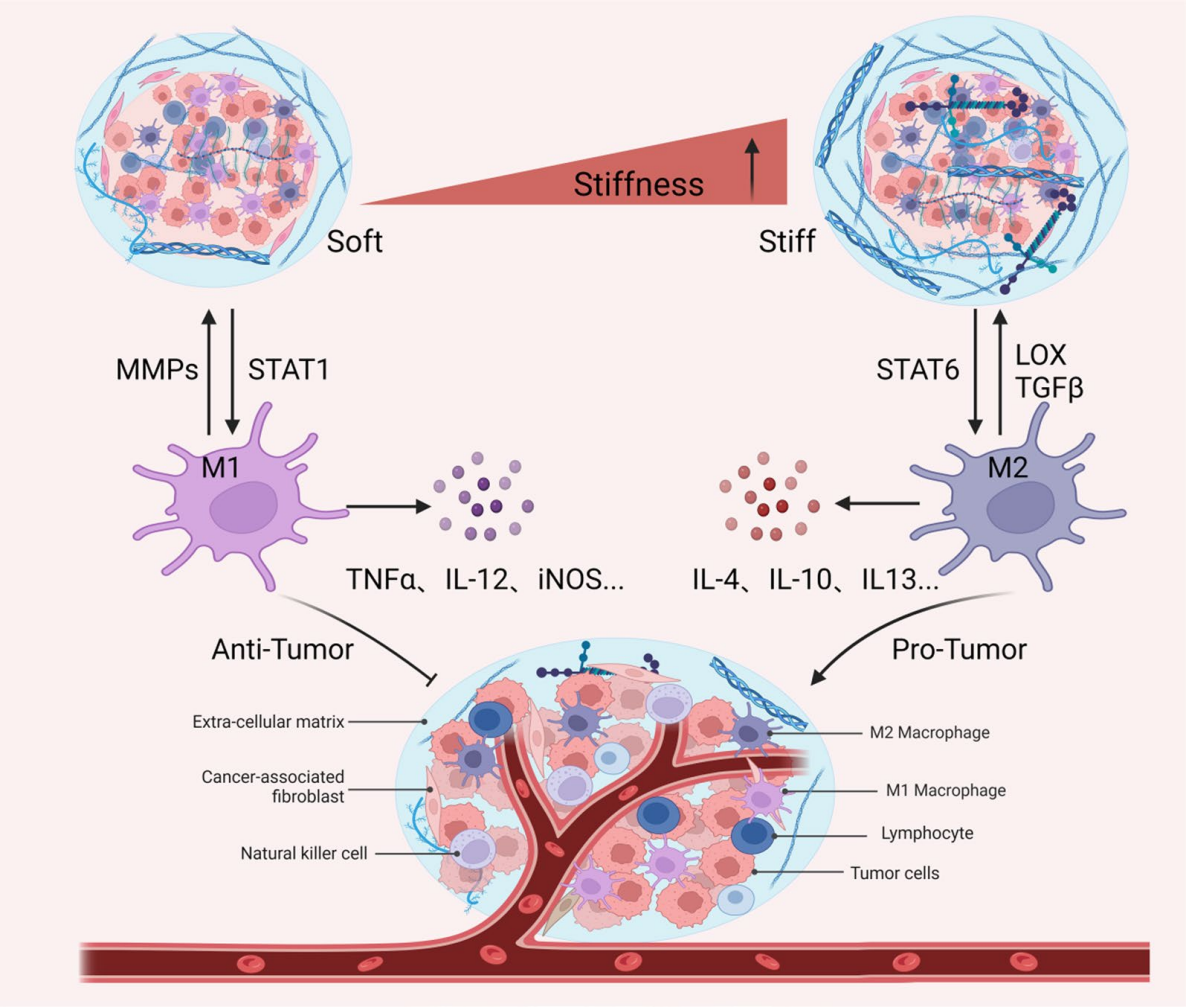
The relationship between matrix stiffness and macrophage polarization in the TME. With the deposition of collagen and the increase in matrix stiffness in the TME, stiff ECM can polarize macrophages into tumor-promoting M2-type TAMs, which in turn also promotes the stiffness of the ECM

The excessive secretion and crosslinking of collagen proteins lead to an increase in matrix stiffness, which increases macrophage mechanosensing [27]. In turn, M2 macrophages activate fibroblasts to become myofibroblasts, which secrete large amounts of collagen to promote matrix deposition while also remodeling the ECM by regulating the balance of MMPs and their inhibitors [28]. Therefore, matrix stiffness and macrophages are mutually regulated in a feedforward manner, ultimately mediating tumor invasion and metastasis (Fig. 1).

### Matrix stiffness regulates macrophage polarization

A growing quantity of evidence suggests that enhanced matrix stiffness promotes macrophages to the M2 phenotype. For example, bone marrow-derived macrophages (BMDMs) cultured on stiff hydrogels upregulate the expression of CD206, IL-4 and TGF-β with less production of reactive oxygen species (ROS), while M2 macrophages can convert to the M1 phenotype when BMDMs are cultured on soft hydrogels[29]. In addition, human-derived macrophages exhibit an M2 phenotype in stiffer 3D matrices, as determined by the overexpression of IL-10 cytokines [30], similar to ECM deposition contributing to macrophage M2 polarization in metastatic breast cancer [31]. Likewise, single-cell RNA sequencing was used to assess the effects of matrix stiffness on intratumor heterogeneity between stiff and compliant mouse mammary tumors. The results showed a significantly higher proportion of M2-like macrophages in the stiffer TME [32]. Furthermore, BMDMs and TAMs isolated from murine tumors cultured in high collagen matrices mimicking tumor tissue showed similar expression of immunosuppressive genes and chemokines, which inhibited the chemotaxis and proliferation of cytotoxic CD8^+^ T cells in coculture assays [33].

Consistent with the above viewpoint, cancer-associated fibroblast (CAF)-induced ECM deposition and matrix stiffness increases can promote cancer progression with poor prognosis [27, 34]. It has been reported that CAFs are highly correlated with TAMs, accompanied by high expression of both CAF and TAM markers, such as α-SMA, FAP, and CD163, in patients with worse clinical prognosis [35, 36]. Furthermore, CAFs are able to facilitate monocyte migration into tumors and polarize into the M2 phenotype. For instance, CAF-derived M-CSF1, IL-6, and CCL2 in monocyte recruitment increased the M2/M1 TAM ratio in pancreatic cancer [37]. Similarly, CCL2, IL-8, IL-10, and TGF-β secreted by CAFs have also been shown to promote the recruitment of monocytes and their transformation into M2 macrophages [38, 39]. Furthermore, CAFs are the top secreting factors primarily to TAMs and engage in mutual paracrine interactions with TAMs, as verified by an experimental-mathematical approach in female breast cancer [40]. Together, these results suggest that matrix stiffness may promote macrophage M2 polarization in tumors.

### Macrophages promote extracellular matrix deposition

ECM stiffness can influence the TAM phenotype; in turn, TAMs can also promote tumor invasion and metastasis by remodeling the ECM composition and structure by instructing the degradation, deposition, crosslinking, and linearization of collagen fibers during tumor development [41]. A recent study showed that TAMs can promote fibrosis in pancreatic cancer by mannose receptor-mediated collagen internalization and subsequent lysosomal degradation-induced metabolic reprogramming [42]. Likewise, TAMs advance tumor progression by the synthesis and assembly of collagenous ECM, specifically collagen types I, VI, and XIV, resulting in the remodeling of its ECM composition and structure in a colorectal cancer mouse model [41]. Similarly, TAMs contribute to tumor progression via legumain-mediated remodeling of ECM deposition and angiogenesis in diffuse large B-cell lymphoma [43]. Interestingly, macrophages mediate the development of fibrosis via the secretion of growth factors and matricellular proteins within obese adipose tissue [44]. In addition, TAMs can fabricate an immunosuppressive network by secreting immunosuppressive factors, mediating the activation of CAFs, and finally leading to ECM deposition [45]. However, when macrophages were engineered with a designed chimeric antigen receptor (CAR), the infused CAR-147 macrophages reduced collagen deposition and promoted T-cell infiltration within HER2-4T1 tumors, which significantly inhibited tumor growth in a BALB/c mouse model [46]. Thus, TAMs have obvious plasticity when inhabiting different environments and can affect tumor progression by remodeling ECM stiffness.

## Matrix stiffness regulates macrophage polarization through mechanoreceptors

**Fig. 2:**
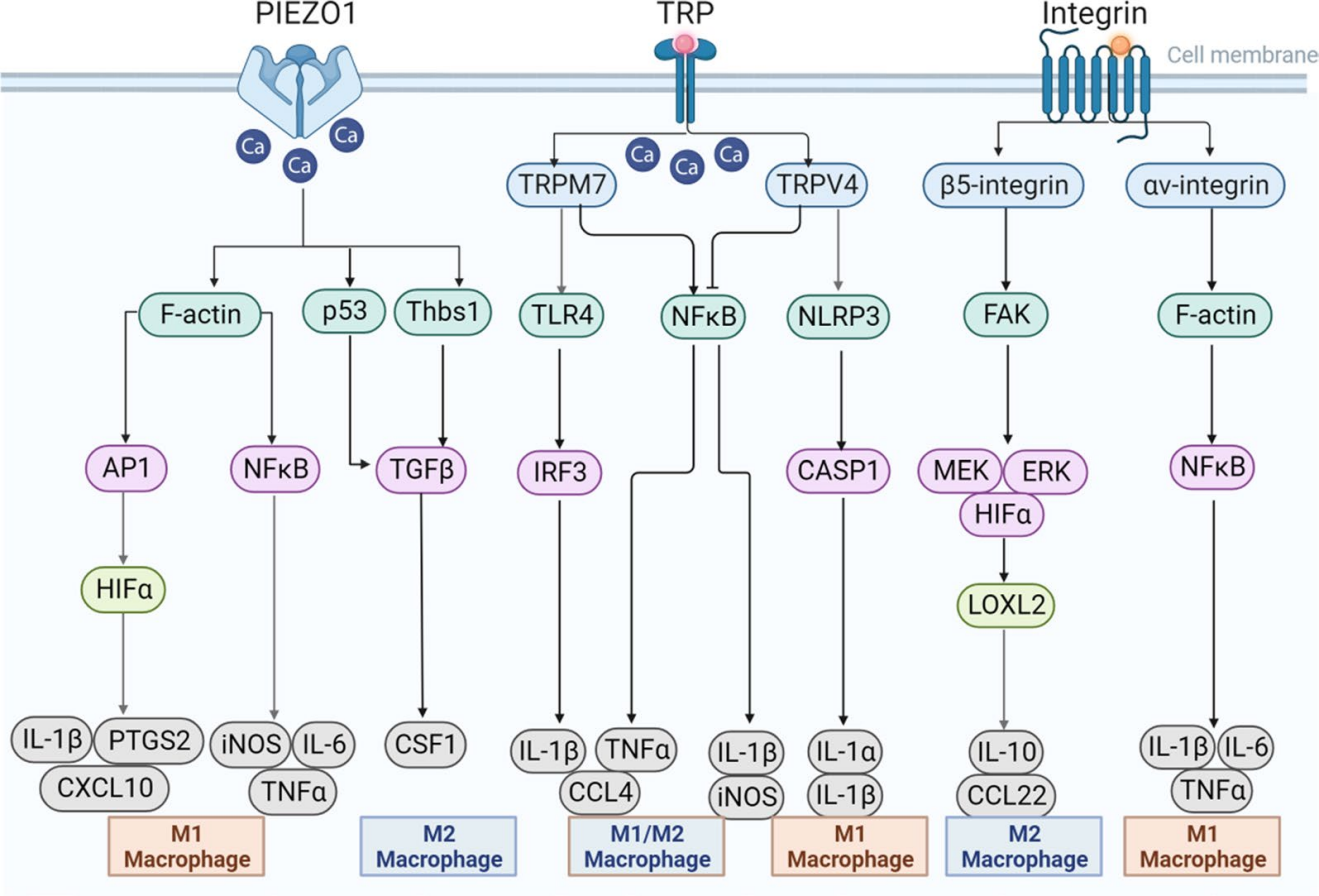
Matrix stiffness regulates macrophage polarization via mechanoreceptors. In different studies, the mechanoreceptors Piezo1, TRP ion channels and integrins on macrophages regulated by matrix stiffness can polarize into M1- or M2-type macrophages

Matrix stiffness shapes cell morphology and function during adult homeostasis. In addition, it also signals to macrophages via mechanoreceptors to affect their polarization and function in the TME. Currently, Piezo1, transient receptor potential (TRP) ion channels, and integrins are the three main mechanoreceptors on macrophages regulated by matrix stiffness or mechanical stimulation, and the regulatory relationship between them is complex (Fig. 2).

### Piezo1

Piezo1, a non-selective Ca^2+^-permeable channel receptor that senses external mechanical forces, can be activated in response to different matrix stiffnesses as well as a variety of mechanical stimuli [47–49].

Macrophages sense and migrate to fibroblast-mediated ECM deposition [50], with subsequent activation of mechanoreceptors such as Piezo1. When BMDMs are mechanically stimulated, they first induce the activation of AP-1 through Piezo1, promoting the expression of endothelin-1 and the stabilization of HIF1α, which in turn promotes the expression of inflammatory cytokines such as IL1β, PTGS2 and CXCL10 [51]. Consistent with this perspective, matrix stiffness-activated ion channel Piezo1 induces macrophage M1 polarization by triggering the NFκB pathway with the upregulation of the pro-inflammatory cytokines of iNOS, TNFα, and IL-6 [52]. Similarly, the complex of Piezo1 and TLR4 together remodeled macrophage F-actin organization and consequently activated the CaMKII-Mst1/2-Rac axis to augment phagocytosis and killing ability [53]. In contrast, mechanical stretch can induce RAW264.7 macrophages toward M2 polarization and TGF-β1 release through the Piezo1 channel to enhance bone formation [54, 55]. In addition, using myeloid cell-specific knockout mice (Lyz2^Cre^; Piezo1^fl/fl^) and transplanting orthotopic KPC cell-derived tumors confirmed that Piezo1 deletion unleashes innate immunity against pancreatic ductal adenocarcinoma [56]. Hence, the effects of the mechanoreceptor Piezo1 on macrophages are complex and multiple, with the ability to regulate both M1 polarization and M2 polarization.

### TRP

TRP ion channels are transmembrane ion channels that allow cations to pass through cell membranes non-selectively. In mammals, the TRP channel family consists of six subfamilies, TRPC, TRPV, TRPM, TRPML, TRPP and TRPA, which participate in the proliferation, invasion, metastasis and angiogenesis of cancer cells and are responsible for a variety of sensory responses, including mechanical force [57, 58]. While increased Ca^2+^ influx through TRP channels, including TRPM2, TRPM7 and TRPC1, is widely confirmed to contribute to macrophage polarization by inflammatory agonists [59–61], the role of matrix stiffness on macrophage function through TRP ion channels remains less explored.

A recent study showed that TRPV4 participates in matrix stiffness-induced macrophage M1 polarization in fibrotic skin tissue in vivo and in vitro, while TRPV4 knockdown reduces M1 markers such as IL-1b and Mcp1 [62]. In addition, cyclic mechanical stretch promotes resident CCR2^−^ cardiac macrophage activation through a TRPV4-dependent pathway, representing a protective population that mediates adaptive cardiac remodeling and survival of the chronically failing heart [63]. Similarly, in pulmonary cystic fibrosis, matrix stiffness activates the phagocytic and bactericidal functions of alveolar macrophages by stimulating TRPC6 overexpression [64]. Conversely, the activation of TRP channels, such as TRPM8 and TRPM7, can promote macrophage M2 polarization with the stimulation of inflammatory factors [65, 66]. At present, although many studies have explored the relationship between TRP channels and macrophage function, only a few studies have explore the regulation of matrix stiffness or mechanical stimulation on the polarization and function of macrophages, and this field deserves further exploration.

### Integrins

Integrins are heterodimeric proteins consisting of one α-chain and one β-chain. Each subunit contains three parts, including a long stalk that senses extracellular signals, a transmembrane helix, and a short intracellular tail that connects with cytoplasmic signaling and cytoskeletal proteins [67]. Integrins have been widely recognized as mechanoreceptor proteins that form a mechanical conduction network, which affects normal physiological behaviors such as cell migration, proliferation and differentiation as well as pathological processes such as cancer and tissue fibrosis [68, 69].

A recent study revealed that fibrotic ECM promotes alveolar macrophage M1 polarization via the integrin-NFκB signaling axis, and the activation effect of macrophages could be abrogated by integrin pan-inhibitors as well as NFκB inhibitors [70]. Consistent with the above results, macrophages cultured in stiffer poly(ethylene glycol) hydrogels show enhanced αV integrin staining with a classical activation phenotype (TNF-α, IL-1β, and IL-6) while cultured in lower stiffness hydrogels with reduced macrophage activation [71]. Similarly, the expression of integrin is reduced when macrophages are cultured on a soft matrix, and the phagocytosis function of macrophages is subsequently restricted [72]. In contrast with the above conclusions, matrix stiffness can also activate macrophages toward the M2 phenotype. To clarify whether matrix stiffness alters LOXL2 expression in macrophages within the TME, THP-1 cells cultured on 6 kPa, 10 kPa, and 16 kPa stiffness substrates were induced by phorbol 12-myristate 13-acetate (PMA) and subsequently treated with IL-4 and IL-13. The results showed that increased matrix stiffness remarkably strengthened macrophage M2 polarization and promoted LOXL2 expression by activating the integrin β5-FAK-MEK1/2-ERK1/2-HIF-1α pathway [73]. Similarly, activating αvβ3 integrin in macrophages by mechanical cues can enhance anti-inflammatory M2 macrophage polarization in a 3D macrophage-ECM hydrogel model [74]. In total, a comprehensive assessment, such as the detection of both M1- and M2- macrophage biomarkers in vivo and in vitro together with multiple detection methods, may provide a more definitive conclusion for the role of integrin in macrophage function.

## Matrix stiffness regulates macrophage polarization through mechanotransducers

**Fig. 3:**
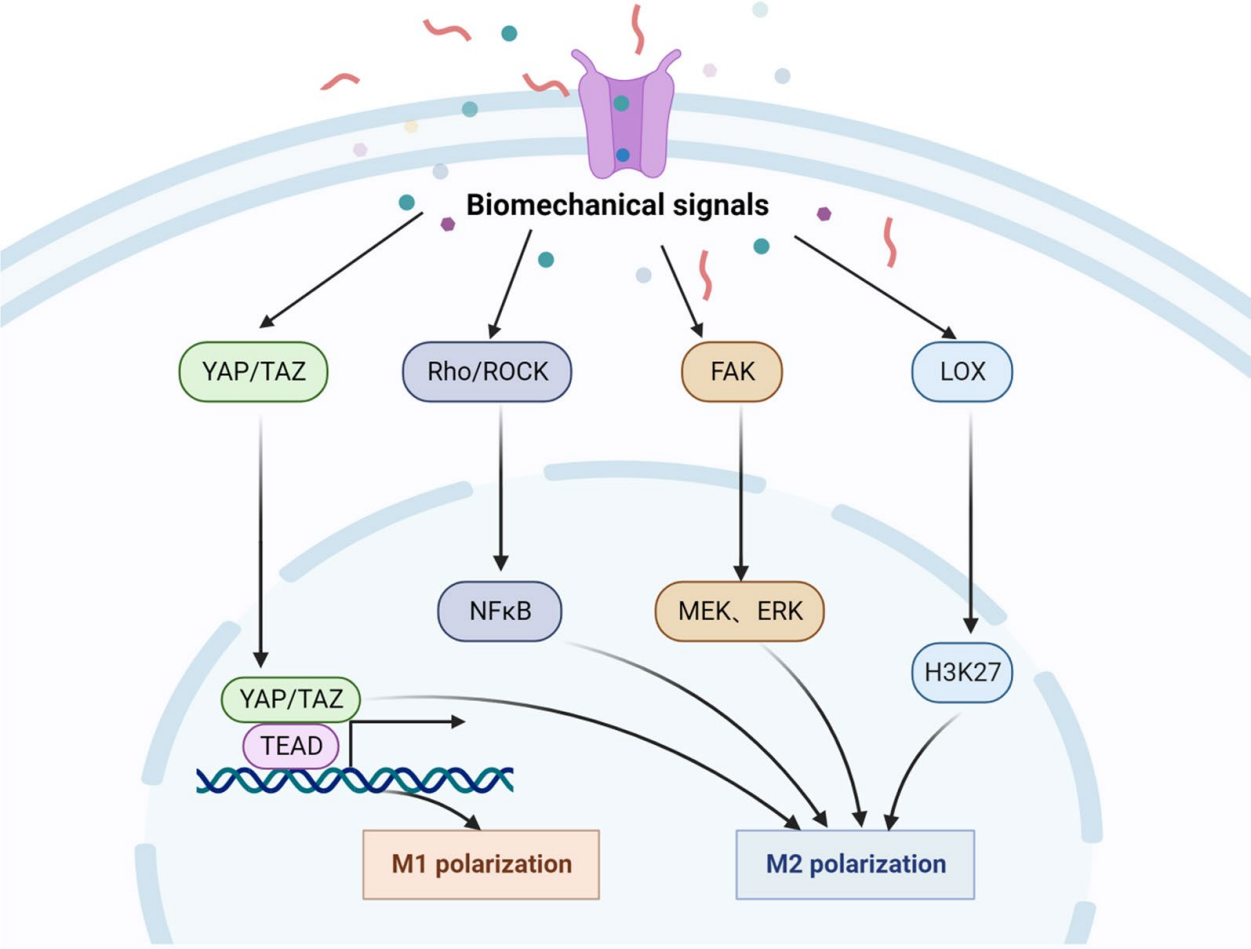
Matrix stiffness regulates macrophage polarization by mechanotransducers. When mechanoreceptors are activated by matrix stiffness, they can transmit mechanical signals to biological signals through different mechanotransducers, thus mediating the polarization of M1- or M2-macrophages by different pathways

Mechanotransducers are key mechanically dependent molecules mediating mechanical signal transduction in the cytoplasm and nucleus. In the TME, YAP/TAZ, Rho/ROCK, FAK, and LOX are the main mechanotransducers that are mostly overexpressed in tumor tissues and regulate macrophage polarization and function stimulated by matrix stiffness and mechanical force (Fig. 3).

### YAP/TAZ

Yes-associated protein (YAP) and transcriptional coactivator with PDZ-binding motif (TAZ) are downstream transducers of the Hippo pathway, engaging in cell proliferation and survival and playing vital roles in controlling organ growth, stem cell self-renewal and cell differentiation [75, 76]. In recent years, accumulating evidences demonstrate that mechanical force has widely been linked to the activity of the transcriptional coactivators YAP and TAZ, establishing a connection between extracellular biomechanics and gene regulation [77, 78].

Macrophage adhesion on soft hydrogel substrates reduces inflammation when compared to that of gels with stiffness, accompanied by decreased YAP expression and nuclear localization [79]. Similarly, macrophages cultured in stiff scaffolds upregulate YAP expression and induce inflammation and doxorubicin drug resistance in osteosarcoma [80]. In another study, low ECM protein expression and reduced YAP activation were accompanied by an increase in M1 macrophages, showing a favorable clinical prognosis in uterine sarcomas [81]. In stiff breast cancer tissue, YAP also induces macrophage M2 polarization and subsequently inhibits CD8^+^ T-cell activity and promotes tumor progression [82]. Furthermore, macrophage M2 polarization was increased in colorectal cancer by activating the Rho/Hippo/YAP signaling pathway [83]. Taken together, matrix stiffness can shape macrophage polarization and function through YAP/TAZ mechanotransduction molecules in the cancer microenvironment.

### Rho/ROCK

The Ras homolog family (Rho) and Rho-associated coiled-coil containing protein serine/threonine kinase (ROCK) signaling pathways are involved in a variety of key biological processes including the regulation of the cytoskeleton and morphogenesis, which shape the TME [84, 85]. Generally, the Rho GTPase family can be divided into three classes: Rho (RhoA, RhoB, and RhoC), Rac (Rac1, Rac2, and Rac3), and cell division cycle 42 (Cdc42), while the ROCK family includes two members, ROCK1 and ROCK2 [86–88]. There is accumulating evidence that the Rho-ROCK pathway is engaged in ECM stiffness and composition, which ultimately promotes the growth, migration, and invasion of cancer cells [89].

Studies from Tu et al. suggest that mechanical stretch promotes RAW264.7 macrophage polarization and inflammatory secretion via the activation of the RhoA-ROCK-NF-κB pathway [90]. Moreover, to explore the effect of different polyacrylamide gel stiffnesses on macrophage polarization state and function, soft (11 kPa), medium (88 kPa), and stiff (323 kPa) gels were constructed, and the results showed that the substrate stiffness-mediated Rho-ROCK pathway plays an important role in directing macrophage behavior [91]. Similarly, pirfenidone treatment can disrupt the polarization and mechanical activation of macrophages by suppressing ROCK2 protein expression (92). In addition, monocyte and macrophage migration into tumor tissues requires the rearrangement of their actin cytoskeleton and is confirmed to be mediated by ROCK, as the ROCK inhibitor Y-27632 contributes to decreased macrophage infiltration in breast tumor tissue [93]. Likewise, examining the influence of ECM (composition, architecture, and stiffness) on the 3D migration of human macrophages demonstrated that macrophages migrate into tissues using either the protease-dependent mesenchymal migration mode or the Rho-ROCK-mediated amoeboid migration mode [94]. Therefore, ECM stiffness can widely affect macrophage behavior via the Rho-ROCK pathway.

### FAK

Focal adhesion kinase (FAK) is a cytoplasmic non-receptor protein tyrosine kinase belonging to the protein tyrosine kinase superfamily. FAK plays crucial roles in cell signal transduction, receives signals from integrins, growth factors and mechanical stimulation, activates intracellular PI3K/Akt, Ras/MAPK and RAS/RAF/MEK pathways, and is related to tumorigenesis and migration [95, 96].

Stiffer polydimethylsiloxane substrates accelerated BMDM and RAW264.7 macrophage osteoclast differentiation by activating the cytoskeleton-associated adhesion molecules fibronectin and integrin αvβ3 and subsequently the biochemical signaling cascades of FAK, PKC, and RhoA [97]. In addition, matrix stiffness-mediated HIF-1α overexpression promotes THP-1-derived macrophage M2 polarization by activating the integrin β5-FAK-MEK1/2-ERK1/2 pathway [73]. Furthermore, the excessive secretion of S100A7 protein stimulated by stiff esophageal squamous carcinoma promotes macrophage M2 polarization and angiogenesis through the activation of the p-FAK and p-ErK pathways [98]. However, by using a Flexcell Tension system, RAW264.7 macrophages promoted M1 polarization-related gene expression and cytokine release after mechanical stretch, and mechanically stretch-preconditioned RAW264.7 cells showed a tumoricidal effect on melanoma in vitro and in vivo [99]. In addition, increased extracellular pressure during infection or inflammation promotes THP-1-derived macrophage phagocytosis by inhibiting FAK expression [100]. These results suggest that FAK can sense stiffness to regulate macrophage function.

### LOX

Lysyl oxidase (LOX) family members, including LOX, LOXL1, LOXL2, LOXL3, and LOXL4, are extracellular copper-dependent enzymes that play a crucial role in ECM crosslinking, which is relevant to fibrosis and oncogenesis [101]. The crosslinking of ECM components, especially collagens and elastin, is strongly engaged in collagen deposition and matrix stiffness [102].

Macrophage-secreted oncostatin M induces LOXL2 expression and ECM collagen remodeling, which promotes primary and metastatic tumor progression and decreases overall survival (OS) in KRas^G12D^-driven pancreatic ductal adenocarcinoma [103]. Furthermore, macrophages drive stromal cell-dependent collagen crosslinking and stiffening promoting breast cancer aggression due to the high expression of LOX [104]. Moreover, LOXL4 promotes macrophage infiltration and matrix deposition, followed by the activation of an immunosuppressive M2 phenotype and programmed death ligand 1 (PD-L1) expression, which further suppress the function of CD8^+^ T cells in hepatocellular carcinoma [105]. During breast tumorigenesis, the EZH2-miR-29b/miR-30d-LOXL4 signaling pathway is activated accompanied by the infiltration of macrophages [106]. In addition, epigenetic regulation of LOX plays an important role in tumor progression; for instance, LOX derived from M2-like macrophages promoted breast cancer cell migration and collagen crosslinking, and this phenomenon was suppressed by an H3K27 demethylase inhibitor [107]. These results suggest that LOX-induced matrix stiffness is involved in M2 macrophage polarization.

## Drugs targeting matrix stiffness for tumor therapy

**Fig. 4:**
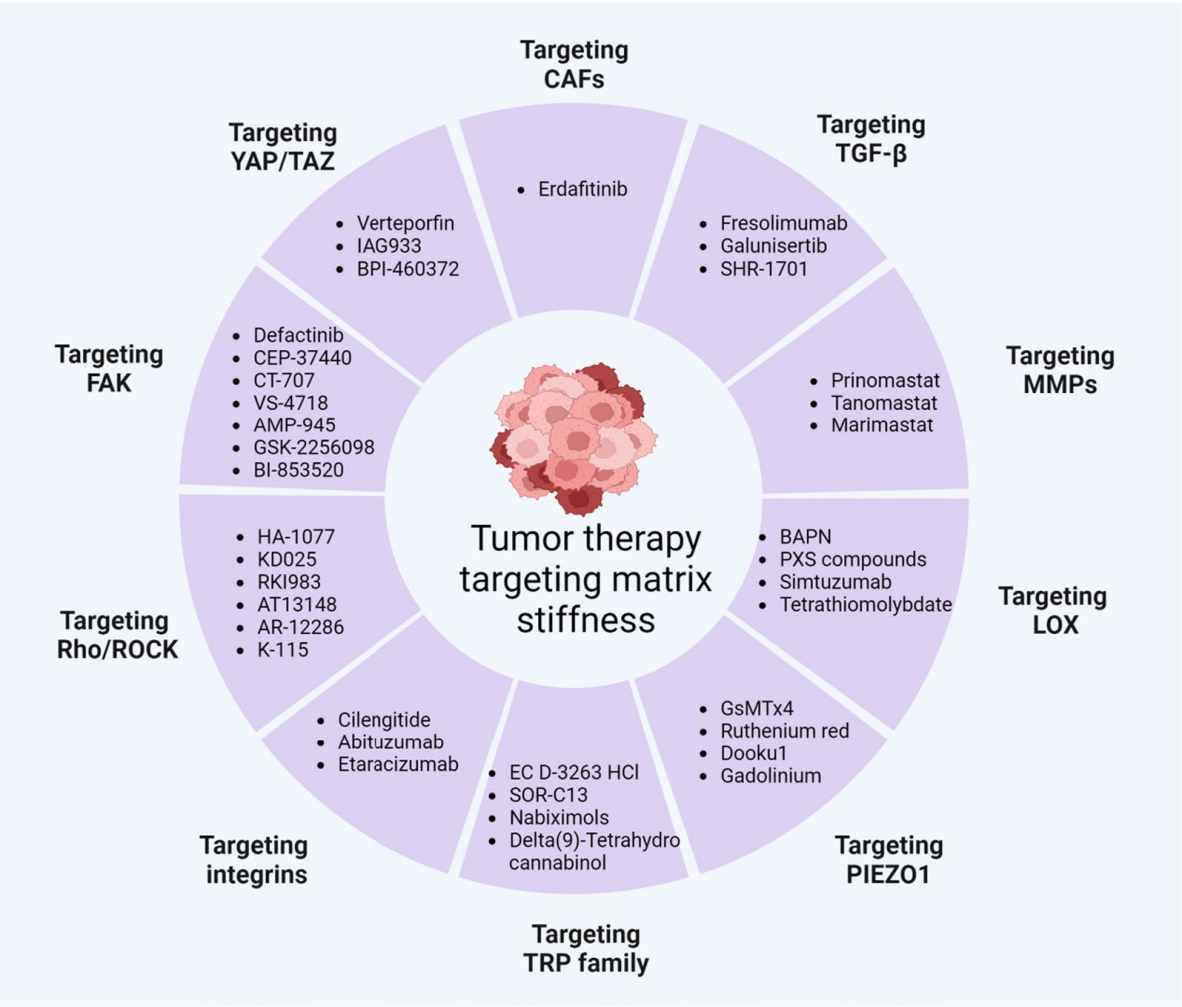
Drugs targeting matrix stiffness for tumor therapy

Since matrix stiffness can affect macrophage function and promote tumor progression in the TME, targeting matrix stiffness can promote macrophages toward an antitumor phenotype and may be a new strategy for cancer treatment [108]. In addition, targeting matrix stiffness can also anchor other stromal cells and delay tumor progression in macrophage-dependent and macrophage-independent ways [109]. At present, three main strategies can be used to interfere with the effect of matrix stiffness on tumor progression: [1] reducing matrix protein production, [2] degrading matrix protein and crosslinking, [3] targeting mechanoreceptors and mechanotransducers stimulated by matrix stiffness (Fig. 4, Table 1).

**Table 1:** The clinical trials of drugs targeting CAFs, TGF-β, MMPs, LOX, TRP, Integrins, Rho/ROCK, FAK and YAP1/TAZ for cancer therapy

Target	Drug	Drug properties	Tumor type	Phase	enrollment	Status/results	Trial ID
CAFs	Erdafitinib	Pan-FGFR inhibitor	Advanced solid tumors	Phase 2	316	Recruiting	NCT04083976
			Bladder cancer	Phase 2	25	Recruiting	NCT04917809
			Advanced solid tumors	Phase 2a	35	Recruiting	NCT02699606
			Prostate cancer	Phase 2	9	poorly tolerated	ACTRN12618001061224
			Advanced NSCLC	Phase 2	22	Completed	NCT03827850
TGF-β	Fresolimumab	Neutralizing antibody of TGF-β	Metastatic Breast Cancer	Phase 2	23	Favorable prognosis	NCT01401062
			Malignant pleural mesothelioma	Phase 2	13	Favorable prognosis	NCT01112293
	Galunisertib	TGF-β receptor kinase inhibitor	Solid tumors	Phase 1b/2	170	Favorable prognosis	NCT01373164
			Solid tumors	Phase 1b/2	25	Well tolerated	NCT02423343
			Hepatocellular Carcinoma	Phase 2	47	Favorable prognosis	NCT01246986
			Solid tumors	Phase 1b	26	Recruiting	NCT03206177
	SHR-1701	Anti-PD-L1/TGF-βRII	Advanced colorectal cancer	Phase 2/3	NA	Recruiting	CTR20210880
MMPs	Prinomastat	Pan-MMPs inhibitor	Advanced NSCLC	Phase 3	362	Failure	NCT00004199
	Tanomastat	Biphenyl MMP inhibitor	Advanced ovarian cancer	Phase 3	243	Failure	NCIC-CTG trial OV12
	Marimastat	Pan-MMPs inhibitor	Metastatic breast cancer	Phase 3	179	Failure	ECOG-E2196
LOX	Simtuzumab	Neutralizing antibody of LOXL2	Pancreatic cancer	Phase 2	240	Failure	NCT01472198
			Colorectal Adenocarcinoma	Phase 2	249	Failure	NCT01479465
	Tetrathiomolybdate	LOX inhibitor	Prostate Cancer	Phase 2	19	Failure	NCT00150995
			Esophageal cancer	Phase 2	69	Well tolerated	NCT00176800
TRP	EC D-3263 HCl	TRPM8 agonist	Advanced solid tumors	Phase 1	23	Completed	NCT00839631
	SOR-C13	TRPV6 antagonist	Advanced solid tumors	Phase 1	23	Well tolerated	NCT01578564
	Nabiximols	TRPV2 agonists	Recurrent glioblastoma	Phase 1b	12	Favorable prognosis	NCT01812616
Integrins	Cilengitide	αvβ3 and αvβ5 integrin inhibitor	Glioblastoma	Phase 3	545	Failure	NCT00689221
	Abituzumab	αv integrin inhibitor	Prostate cancer	Phase 2	180	Failure	NCT01360840
	Etaracizumab	Neutralizing antibody of αvβ3 integrin	Metastatic melanoma	Phase 2	112	Failure	NCT00066196
Rho/ROCK	AT13148	ROCK-AKT inhibitor	Solid tumors	Phase 1	51	Failure	NCT01585701
FAK	Defactinib	FAK inhibitor	Advanced NSCLC	Phase 2	55	Well tolerated	NCT01951690
			Pleural Mesothelioma	Phase 2	344	Failure	NCT01870609
	Conteltinib	FAK inhibitor	Advanced NSCLC	Phase 1	60	Favorable prognosis	NCT02695550
YAP/TAZ	Liposomal Verteporfin	YAP1 inhibitor	Glioblastoma	Phase 1/2	24	Recruiting	NCT04590664
	IAG933	TEAD inhibitor	Solid Tumors	Phase 1	156	Recruiting	NCT04857372
	BPI-460372	TEAD inhibitor	Solid Tumors	Phase 1	82	Recruiting	NCT05789602

*NCIC-CTG trial* Standard National Cancer Institute of Canada-Clinical Trials Group, *ECOG-E2196* Eastern Cooperative Oncology Group trial E2196, *ACTRN* Australian New Zealand Clinical Trials Registry, *CTR* China drug trials

### Reducing matrix protein production

CAFs and TGF-β are the main factors leading to the production of matrix proteins in the TME. To reduce matrix protein production and matrix stiffness, targeting CAFs and TGF-β has achieved great attractions in preclinical and clinical studies.

#### Targeting CAFs

CAFs actively participate in tumor progression through complicated interactions with other types of stromal cells and produce ECM components that contribute to the remodeling of the tumor stroma [39, 110]. An array of CAF biomarkers have been identified, including but not limited to αSMA, FSP1, FAP, PDGFRα, PDGFRβ, CLEC3B, Desmin, DDR2 and Vimentin, and many therapeutic approaches targeting CAFs have been well reviewed [111–113]. CAFs have long been considered an attractive therapeutic target with the majority of studies showing tumor-promoting roles of CAFs [114, 115].

At present, erdafitinib, a pan-FGFR inhibitor, has achieved expedited approval from the US Food and Drug Administration (FDA) for locally advanced or metastatic urothelial carcinoma in patients with FGFR alterations due to the positive objective tumor response in a phase 2 clinical trial [116]. However, erdafitinib has obvious adverse events in clinical trials with poor tolerability, and the antitumor effect of erdafitinib should be further anticipated in subsequent studies [116, 117]. In contrast, in some preclinical studies, targeting the elimination of CAFs even promotes tumor progression and results in poor prognosis [118, 119]. Moreover, targeting CAFs-related signaling pathways, such as SHH–SMO signaling or hyaluronic acid, even shortened patient survival in clinical trials [120–122]. Recently, single-cell analysis techniques revealed distinct CAF subpopulations compared to conventional CAF subpopulations [123, 124]. Therefore, we should systemically define the functional roles of CAFs and CAF subpopulations in future studies to comprehensively develop accurate diagnostic and therapeutic approaches based on specific CAF subpopulations.

#### Targeting TGF-β

TGF-β is a subtype of the TGF-β family that plays a crucial role in fibrosis and solid tumors [125]. TGF-β promotion of tumor progression is multifaceted, including altering the polarization of macrophages, damaging the activities of tumor-infiltrating lymphocytes (TILs), and inducing regulatory T (Treg) cell differentiation in the TME [126]. Moreover, TGF-β hinders TIL penetration by increasing peritumoral collagen production [127]. In addition, TGF-β-induced phosphorylation of Smad2/3 also promotes the deposition of α-SMA, MMP1, and collagen type I, which increase matrix stiffness by the overexpression of LOXL1 [128].

At present, several drugs have shown promising results in clinical trials. For example, fresolimumab is a neutralizing antibody targeting all human isoforms of TGF-β and has demonstrated longer median OS in metastatic breast cancer when combined with local radiotherapy [129]. In addition, fresolimumab treatment decreases biomarkers of thrombospondin-1 and cartilage oligomeric protein and improves clinical symptoms in systemic sclerosis patients [130]. Galunisertib is a TGF-β receptor kinase inhibitor that selectively blocks TGF-β signaling and results in the improvement of OS in pancreatic cancer patients when combined with gemcitabine therapy [131]. Similarly, the combination of galunisertib and sorafenib demonstrated acceptable safety and prolonged OS in patients with advanced hepatocellular carcinoma [132]. Other drugs, such as SHR-1701 (bifunctional anti-PD-L1/TGF-βRII agent) and ACE-536 (TGF-β superfamily ligand trap), have reached phase 3 clinical trials (CTR20210880, NCT04064060, NCT04717414) with promising potential. Although TGF-β pathways contribute to the pathological processes of fibrosis and tumor progression, the physiological functions and side effects of TGF-β cannot be ignored, and the dosing and drug delivery systems of TGF-β-based therapies need further study in the future.

### Matrix protein degrading and crosslinking

To reduce the matrix stiffness of the cancer environment, degrading matrix proteins and crosslinking is another good strategy in addition to the elimination of matrix protein production. Therefore, targeting MMPs and LOX has been conducted in clinical trials for cancer therapy.

#### Targeting MMPs

MMPs are zinc-dependent endopeptidase enzymes that can degrade various ECM proteins [133]. The initial clinical trials were designed based on the theory that ECM provides a biological barrier preventing endothelial cell migration or tumor cell invasion, and activated MMPs can degrade ECM, helping cancer cells break through the matrix barrier and promoting the invasion and metastasis of malignant tumors [134, 135]. Due to the high expression of MMPs in metastatic tissues, targeting MMP may be a viable solution. However, phase 3 clinical trials of inhibitors (Prinomastat, Tanomastat, Marimastat) targeting MMPs have all ended in failure [136–138]. Thus, to achieve better cancer therapy targeting MMPs, a comprehensive understanding of the roles of MMPs in cancer progression must be evaluated in depth.

#### Targeting LOX

The LOX family of enzymes is responsible for matrix remodeling by covalent crosslinking of collagen and elastin at primary and metastatic tumor sites [139]. Due to the high expression of LOX in most types of human cancers, LOX family members have emerged as potential clinical targets for cancer therapy [140].

Until now, an array of LOX family inhibitors, such as BAPN, PXS compounds, tetrathiomolybdate, CCT365623, PAT-1251, AB0023 and simtuzumab, have been developed for tumor therapy. BAPN is the first pan-inhibitor of the LOX family with the features of non-specificity and irreversibility [141]. Although BAPN showed an antitumor effect by suppressing LOX activity in many preclinical studies [142–144], no clinical trials have been conducted for BAPN due to the lack of suitable chemical modification sites as well as the non-tumorigenic toxicity and teratogenic effect [139, 145, 146]. PXS compounds are a new generation of oral pan-LOX inhibitors, including PXS-S1A, PXS-S2A, PXS-S1C, PXS-5153A, and PXS-5505. Among these, PXS-5505 is being tested in clinical trials for hepatocellular carcinoma (NCT05109052) and thrombocythemia myelofibrosis (NCT04676529). In addition, AB0023, a neutralizing antibody against LOXL2, can inhibit the activity of LOXL2, reduce activated fibroblasts, and inhibit tumor progression [147]. Similarly, simtuzumab is a humanized monoclonal antibody of AB0023, and has been engaged in phase 2 clinical trials for pancreatic adenocarcinoma and metastatic KRAS mutant colorectal adenocarcinoma, whereas, both studies ended in failure [148, 149]. A possible explanation is that although simtuzumab can prevent further collagen crosslinking, it cannot reverse the crosslinks already present in the ECM. Thus, simtuzumab may be effective in the early stage of malignancy.

### Targeting mechanoreceptors and mechanotransducers

To interfere with matrix stiffness-induced signaling, mechanoreceptors and mechanotransducers may be alternative targets in addition to the elimination of CAFs and TGF-β as well as regulators of collagen deposition and crosslinking. Therefore, targeting Piezo1, the TRP family, integrins, FAK, Rho/ROCK, and YAP/TAZ has gained great interest in recent years.

#### Targeting Piezo1

Piezo1 is a cationic mechanical receptor that is widely regulated by matrix stiffness or mechanical stimulation and is involved in the progression of tumors [150]. Most studies report that Piezo1 is highly expressed in different types of tumors and is associated with poor prognosis [151, 152]. For example, Piezo1 activates integrin-FAK signaling, regulates ECM and reinforces tissue stiffness to promote glioma aggression, while deleting Piezo1 inhibits tumor aggression and prolongs survival [153]. At present, only a few Piezo1 inhibitors (GsMTx4, ruthenium red, Dooku1, gadolinium) have been reported in preclinical studies and no agents are engaged in clinical trials [56, 154–156]. The role of Piezo1 inhibitors in tumors is still in the initial stage, and with more in-depth research in the future, the development of drugs targeting Piezo1 may be of great value.

#### Targeting the TRP family

TRP family ion channels are other types of mechanoreceptors that are widely studied in the sensation of coldness, heat, pain, taste and vision as well as mechanic stimulation. Among them, TRPV, TRPM, and TRPC subfamily members are mostly studied and associated with malignant growth and progression [157], and most of them are overexpressed in different kinds of cancers such as breast cancer, pancreatic cancer, glioblastoma, ovarian cancer, lung cancer, and colon cancer [158–160]. In contrast, some studies have found that TRPV4 activation in endothelial cells can inhibit tumor angiogenesis, growth and metastasis by suppressing the expression of VEGFR2, p-ERK, and MMP-9 [161]. In addition, activation of TRPV4 also inhibits the progression of glioma, melanoma, and breast cancer [162–164]. At present, most of these results are evaluated in preclinical studies, and a few studies have been conducted in clinical trials when treated with tumors.

D-3263 hydrochloride is an enteric-coated, orally bioavailable TRPM8 agonist and is being tested in a phase 1 clinical trial in patients with advanced solid tumors [NCT00839631]. Similarly, SOR-C13 is a high-affinity antagonist of TRPV6 and was evaluated in a phase 1 clinical trial in advanced solid tumors, suggesting antitumor activity because of stable disease survival [165]. Delta[9]-Tetrahydrocannabinol and nabiximols, nonspecific agonists of TRPV2, were tested in a phase 1 clinical trial in recurrent glioblastoma and both showed antitumor activity [166, 167]. Together, future studies should focus on the exact relationship between the TRP family and tumors to lay a firm theoretical foundation for the design of specific targeted drugs.

#### Targeting integrins

The integrin family consists of 24 transmembrane glycoprotein members, and a large proportion of integrin subfamilies are overexpressed during cancer progression [168, 169]. Integrin-mediated sensing, stiffening and remodeling of ECM stiffness are crucially important for cancer progression, supporting invasion and drug resistance [168]. Therefore, targeting integrins may reverse the effects of ECM stiffness on tumorigenesis.

Currently, approximately 90 kinds of integrin-based therapeutic agents or imaging drugs have been used in clinical studies, among which 16 drug types, including cilengitide, antiangiotide, etaracizumab, volociximab, intetumumab, abituzumab, BGC-0222, ProAgio, CEND-1, HYD-PEP-06, 7HP-349, ATN-161, SGN-B6A, ABBV-382, OPC-415, and MT-1002, have been reported to target integrin subfamilies of αv, α5β1, αvβ3, αLβ2, α4β1, β6, α3β1, β1, and β7 in clinical trials for cancer treatment [170]. For preclinical research, targeting integrins is quite mature with remarkable antitumor effects [171–173]. However, most previous clinical trials targeting integrins are disappointing, even in the phase 3 clinical trial of cilengitide for treating glioblastoma [174–176]. Despite the disappointing outcome of targeting integrins in clinical studies, integrins play an important role in tumorigenesis, and further mechanistic research should be conducted between integrins and tumors.

#### Targeting Rho/ROCK

The Rho-ROCK signaling pathway participates in a variety of key biological processes, including cytoskeleton remodeling, ECM stiffness and cancer progression [85]. In most solid tumors, such as pancreatic cancer, breast cancer, renal cancer, urothelial cancer and osteosarcoma, the Rho-ROCK signal is overexpressed and activated and is often associated with shortened OS [177–179]. However, the research of Rho-ROCK signaling in malignant tumors is still insufficient and lacks major breakthroughs. Although some Rho/ROCK inhibitors, such as fasudil (HA-1077), KD025, RKI983, AT13148, verosudil (AR-12286), ripasudil (K-115), have been studied in clinical studies for other diseases, currently, only a few Rho-ROCK inhibitors have been conducted in clinical trials for cancer therapy. For instance, AT13148, an oral dual ROCK-AKT inhibitor, failed in a phase 1 clinical trial in patients with solid tumors due to the narrow therapeutic index and pharmacokinetic profile [180]. Therefore, future works should further clarify the roles of Rho-ROCK in tumors.

#### Targeting FAK

FAK is a nonreceptor tyrosine kinase that is upregulated in a wide variety of solid cancers (such as head, neck, oral, thyroid, lung, breast, bladder, colorectal, prostatic, hepatocellular carcinomas and ovarian cancer) [181]. Therefore, FAK can be considered a potential target for cancer therapy.

To date, a large number of inhibitors have been designed based on the crystallographic structures of FAK kinase domains such as defactinib, CEP-37440, conteltinib (CT-707), VS-4718, AMP-945, GSK-2256098 and BI-853520, which were all engaged in phase 1/2 clinical trials for cancer therapy [182]. Currently, most clinical studies are in the early stage, and its antitumor effect needs to be verified in subsequent studies. In addition, other attractive FAK inhibitors, such as TAE226, PF-573228 and Y15, were tested in preclinical studies with significant antitumor effects [183–185]. At present, although no compounds have been launched on the market, there is great interest in the development of novel FAK inhibitors, which represent a new emerging therapeutic method for cancer treatment.

#### Targeting YAP/TAZ

The YAP/TAZ pathway can be activated by mechanical cues such as matrix stiffness and cell stretch, and has been shown to promote tumor growth and invasion in multiple human cancers [186]. Although the majority of studies reveal that YAP/TAZ activation is associated with tumor-promoting effects [187–190], accumulating evidence also demonstrates that YAP/TAZ play vital roles in tumor-suppressive effects in human cancers.

YAP inhibits breast cancer growth by disrupting a TEAD-ERα signaling axis [191, 192]. In addition, YAP inhibits HIF-2α and renal cell carcinoma progression by disrupting the HIF-2α/TEAD signaling complex [193]. Furthermore, YAP1 and WWTR1 expression inhibits the tumor progression of Merkel cell carcinoma [194]. One possible reason for the different conclusions mentioned above may be that TEAD complexes target different enhancers in YAP-low and YAP-high expression cancers [195]. Currently, due to the special structural properties of YAP and TAZ, it is not easy to directly target YAP/TAZ proteins. Therefore, targeting the YAP/TAZ-TEAD complex is a better strategy. Verteporfin, an inhibitor of YAP1, has been shown to inhibit the interaction between YAP1 and TEAD [196], and a phase 1/2 study of visudyne (liposomal verteporfin) in patients with recurrent high-grade EGFR-mutated glioblastoma is recruiting [NCT04590664]. IAG933 and BPI-460372, two kinds of TEAD inhibitors disturbing the YAP-TEAD association, are in the recruitment stage of phase 1 clinical trials for the treatment of patients with advanced mesothelioma and other solid tumors [NCT04857372, NCT05789602]. Other targeting YAP/TAZ-TEAD inhibitors, such as flufenamic acid, TED-347, VGLL4-mimicking peptide, have been used in preclinical studies to evaluate their antitumor effects. Taken together, the effect of YAP/TAZ on various cancers is complex, and inhibitors targeting the YAP/TAZ-TEAD complex need to be further evaluated in clinical studies.

## Conclusions and perspectives

Exploring phenotype, function and mechanism links between ECM stiffness and macrophages in the TME will improve the current knowledge of cancer diagnosis, prognosis, and treatment strategies. By using multiple immunohistochemistry, spatial metabolomics, single-cell analysis techniques, and organoids culture together with flow cytometry analysis for tumors of different rigidities, we may capture more comprehensive and realistic conclusions of TAM status in vivo and may provide correct guidance for targeting therapy based on ECM stiffness. It is important to note that matrix stiffness not only directly affects TAM function but also indirectly regulates the TAM phenotype through other types of cells, especially tumor cells and CAFs [197]. When discussing the effect and mechanism of matrix stiffness on the regulation of macrophages, different kinds of biological materials and scaffolds, such as nanoparticles, fibers, hydrogels, and 3D printing with diverse components, have been used to simulate ECM stiffness and often contribute to inconsistencies in experimental results in vitro and in vivo. This phenomenon may be attributed to the large difference between simulated ECM and natural ECM as well as the different materials used in the experiments. At present, more in-depth research is needed to determine the impact of ECM stiffness on TAM function.

Macrophages have traditionally been divided into the pro-inflammatory M1 phenotype and the anti-inflammatory M2 phenotype. However, macrophage classification is complex in reality, and sometimes it is difficult to evaluate the macrophage phenotype based only on common M1- and M2 biomarkers when both types of biomarkers show a consistent trend of change.Currently, no studies have systematically analyze this situation and defined the classification of macrophages when discussing their phenotype and function based on these biomarkers. How to assess the weighted value of both kinds of biomarkers, whether negative feedback regulation leads to consistent trends or only macrophage biomarkers combined with functional detection, is the gold standard to determine macrophage phenotype. Future work requires more robust evidence to unify the consensus of macrophage classification.

ECM stiffness is widely considered an adverse factor that promotes cancer progression by remodeling stromal cells such as TAMs and CAFs. Therefore, targeting ECM stiffness or mechanical cues is an attractive alternative option for cancer therapy. Therefore, reducing matrix protein production by eliminating CAFs and TGF-β, degrading matrix proteins and crosslinking by blocking the enzyme's action of MMPs and LOX, or targeting mechanoreceptors such as Piezo1, TRP, integrins, and mechanotransducers such as Rho/ROCK, FAK, YAP/TAZ, is gaining increasing attention in preclinical and clinical research for cancer therapy. Unfortunately, no drugs (except for erdafitinib) are approved for the above targets of cancer therapy on the market. It seems that one possible reason for this situation is that degradation of matrix proteins can reduce matrix stiffness, which is beneficial to cancer treatment. However, a stiff matrix is also a physical barrier that prevents the invasion and metastasis of cancer cells. Understanding and balancing these contradictory effects is a key problem that urgently needs to be addressed. Taken together, ECM is an extremely complex system, and in the future, the abovementioned core issues need to be well addressed to provide an optimal strategy for cancer therapy.

## Data Availability

All data generated or analyzed are included in this published article.

## Abbreviations

ECM: Extracellular matrix
TAMs: Tumor-associated macrophages
TME: Tumor microenvironment
MMPs: Matrix metalloproteinases
BMDMs: Bone marrow-derived macrophages
CAFs: Cancer-associated fibroblasts
CAR: Chimeric antigen receptor
TRP: Transient receptor potential
PMA: Phorbol 12-myristate 13-acetate
YAP: Yes-associated protein
TAZ: Transcriptional coactivator with PDZ-binding motif
Rho: Ras homolog family
ROCK: Rho-associated coiled-coil containing protein serine/threonine kinase
Cdc42: Cell division cycle 42
FAK: Focal adhesion kinase
LOX: Lysyl oxidase
OS: Overall survival
PD-L1: Programmed death ligand 1
FDA: Food and Drug Administration
TILs: Tumor-infiltrating lymphocytes
Treg: Regulatory T
CT-707: Conteltinib
Visudyne: Liposomal Verteporfin
